# Draft Genome Sequence of the Moderately Halophilic Bacterium *Marinobacter lipolyticus* Strain SM19

**DOI:** 10.1128/genomeA.00379-13

**Published:** 2013-06-27

**Authors:** R. Thane Papke, Rafael R. de la Haba, Carmen Infante-Domínguez, Dolores Pérez, Cristina Sánchez-Porro, Pascal Lapierre, Antonio Ventosa

**Affiliations:** Department of Molecular and Cell Biology, University of Connecticut, Storrs, Connecticut, USAa; Department of Microbiology and Parasitology, Faculty of Pharmacy, University of Sevilla, Sevilla, Spainb; Center for Medical Science, New York Department of Health, Albany, New York, USAc

## Abstract

*Marinobacter lipolyticus* strain SM19, isolated from saline soil in Spain, is a moderately halophilic bacterium belonging to the class *Gammaproteobacteria*. Here, we report the draft genome sequence of this strain, which consists of a 4.0-Mb chromosome and which is able to produce the halophilic enzyme lipase LipBL.

## GENOME ANNOUNCEMENT

The genus *Marinobacter*, within the class *Gammaproteobacteria*, family *Alteromonadaceae*, currently includes 33 species that are isolated mainly from saline or hypersaline environments (marine habitats, salterns, etc.) (1, 2). *Marinobacter lipolyticus* is a moderately halophilic bacterium (3) isolated from saline soil in Cádiz, Spain (4), following a screening program that focused on isolating and characterizing halophilic bacteria that are capable of producing hydrolytic enzymes useful for biotechnological applications (5–9). *M. lipolyticus* grows optimally at 7.5% NaCl (range, 1 to 15%), 37°C (range, 15 to 40°C), and pH 7.5 (range, pH 5 to 10). It is able to produce a halophilic lipase, designated LipBL, which was expressed in *Escherichia coli* (3). Characterization of LipBL has demonstrated it to consist of 404 amino acids, with a molecular mass of 45.3 kDa, and to have high sequence identity with class C β-lactamases. Though *M. lipolyticus* is not a thermophile, the maximal temperature activity of LipBL was found to be 80°C, pH 7.0, and without NaCl (it maintained 20% activity in a wide range of NaCl concentrations). The highest activity observed was against short-to-medium-length acyl chain substrates, although it also hydrolyzed olive oil and fish oil (10). LipBL is an interesting enzyme, with potential biotechnological applications due to its high regioselectivity (10, 11). The role of conserved amino acids in the lipolytic activity of this family VIII lipase enzyme has been recently studied (12).

A draft genome sequence of *M. lipolyticus* strain SM19 was obtained using a whole-genome shotgun strategy (13) with an Illumina sequencing system (Illumina, Inc., San Diego, CA), which produced paired-end reads of ~45 bp, with an insert size of 300 bp. The final genome assembly has ~100-fold coverage of the entire genome. All reads were assembled into 35 supercontigs composed of 52 contigs (≥89 bp) using Velvet v1.0 (14). Forty-three (83%) of the contigs were ≥510 bp and ≤683,264 bp (average size, 93,563 bp) and were used to identify open reading frames (ORFs) and provide a functional annotation of predicted proteins, rRNA genes, and tRNA genes. Analysis was automated with Integrative Services for Genomic Analysis (ISGA) (15).

The genome is estimated to contain 4,023,208 bp, with a G+C content of 56.7%, and 3,646 putative ORFs with an average size of 995 bp. The assessed coding density is 90.1%. Furthermore, SM19 contains a single rRNA operon and a total of 46 tRNA genes.

The genome analysis confirms the presence of genes for lipase, amylase, protease, and DNase. Ectoine synthase and bacterial type I/II/III/IV/VI secretion systems were also present.

### Nucleotide sequence accession number.

The *M. lipolyticus* strain SM19 Whole-Genome Shotgun project has been deposited at DDBJ/EMBL/GenBank under the accession no. ASAD00000000. The version described in this paper is the first version.
